# Non-typhoidal *Salmonella* intestinal carriage in a *Schistosoma mansoni* endemic community in a rural area of the Democratic Republic of Congo

**DOI:** 10.1371/journal.pntd.0007875

**Published:** 2020-02-21

**Authors:** Lisette Mbuyi-Kalonji, Barbara Barbé, Gaëlle Nkoji, Joule Madinga, Clémentine Roucher, Sylvie Linsuke, Marie Hermy, Anne-Sophie Heroes, Wesley Mattheus, Katja Polman, Pascal Lutumba, Marie-France Phoba, Octavie Lunguya, Jan Jacobs

**Affiliations:** 1 Department of Microbiology, National Institute for Biomedical Research, Kinshasa, Democratic Republic of the Congo; 2 Department of Clinical Biology, Microbiology Unit, University Hospital of Kinshasa, Democratic Republic of the Congo; 3 Department of Clinical Sciences, Institute of Tropical Medicine, Antwerp, Belgium; 4 Department of Biomedical Sciences, Institute of Tropical Medicine, Antwerp, Belgium; 5 Institute of Health and Society, Université Catholique de Louvain, Brussels, Belgium; 6 Department of Epidemiology, National Institute for Biomedical Research; Democratic Republic of the Congo; 7 Department of Tropical Medicine, University Hospital of Kinshasa, Democratic Republic of the Congo; 8 Department of Microbiology and Immunology, KU Leuven, Leuven, Belgium; 9 Department of Human Bacterial Diseases, Sciensano, Brussels, Belgium; 10 Department of Health Sciences, VU Amsterdam, Amsterdam, the Netherlands; University of Pennsylvania, UNITED STATES

## Abstract

**Background:**

Clinical observations and animal studies have suggested that *Salmonella* intestinal carriage is promoted by concurrent *Schistosoma* infection. The present study assessed association of *Salmonella* intestinal carriage and *Schistosoma mansoni* infection among individuals in a *Schistosoma* endemic area in sub-Saharan Africa.

**Methods:**

From November 2015 to March 2016, a cross-sectional community-wide study was conducted in Kifua II, a rural village in Kongo Central Province, Democratic Republic of Congo. Stool samples were collected and analyzed for *Salmonella* intestinal carriage (culture) and *Schistosoma mansoni* infection (Kato Katz microscopy with determination of egg load). *Salmonella* Typhimurium and Enteritidis isolates were assessed for genetic similarity with blood culture isolates obtained during the same period in a neighboring hospital using multi-locus variable-numbers tandem repeat analysis (MLVA).

**Results:**

A total of 1,108 participants were included (median age 15 years (IQR: 7–36), male-to-female ratio of 1:1.1). The overall prevalence of *Schistosoma mansoni* infection and non-typhoidal *Salmonella* carriage was 51.2% (95% CI: 48.2–54.1) and 3.4% (95% CI: 2.5–4.7) respectively, with 2.2% (95% CI: 1.5–3.2) of participants coinfected. The proportion of *Salmonella* carriage tended to be higher among *Schistosoma mansoni* infected participants compared to non-infected participants but this difference did not reach statistical significance (4.2% versus 2.6%, p = 0.132). However, the proportion of *Salmonella* carriage among participants with a heavy *Schistosoma mansoni* infection was significantly higher compared to those with a light and moderate infection (8.7% versus 3.2%, p = 0.012) and compared to *Schistosoma mansoni* negatives (8.7% versus 2.6%, p = 0.002). The 38 *Salmonella* isolates comprised five and four Enteritidis and Typhimurium serotypes respectively, the majority of them had MLVA types identical or similar to those observed among blood culture isolates.

**Conclusion:**

*Salmonella* intestinal carriage was associated with a heavy intensity of *Schistosoma mansoni* infection. Further studies are needed to address causation.

## Author summary

*Salmonella* intestinal carriage may be facilitated by infection with *Schistosoma mansoni*, which is a flatworm that lives in the small blood vessels of the human intestinal tract. This situation has been described in tropical low and middle income countries, where both pathogens are endemic. The present study aimed to assess the association of *Salmonella* intestinal carriage with *Schistosoma mansoni* infection in Kifua II village, located in the Kongo Central province of the Democratic republic of Congo. We collected stool samples from 1,108 inhabitants of the village and analysed them for *Salmonella* (culture) and *Schistosoma mansoni* eggs (microscopy). Our findings showed that 3.4% of the participants had *Salmonella* in their stool and 51.2% were infected with *Schistosoma mansoni*. There was no association between overall *Schistosoma* infection and *Salmonella* carriage but *Salmonella* carriage was significantly higher among participants with heavy *Schistosoma* infection compared to those with a light and moderate infection (8.7% versus 3.2%, p = 0.012) and compared to those without *Schistosoma mansoni* infection (8.7% versus 2.6%, p = 0.002). Further, we showed that some *Salmonella* isolates recovered from stool were very similar to isolates obtained from invasive infections (blood cultures from patients in a neighbouring hospital).

## Introduction

In sub-Saharan Africa, *Salmonella* is among the most frequent bacteria causing invasive infections in humans [1,2]. Invasive *Salmonella* infections comprise the human-restricted typhoid fever (caused by *Salmonella enterica* subspecies *enterica* serotypes Typhi and Paratyphi) as well as the non-typhoidal *Salmonella* (NTS) infections. Invasive NTS mainly include two serotypes: *Salmonella enterica* serotype Typhimurium (*Salmonella* Typhimurium) and *Salmonella enterica* serotype Enteritidis (*Salmonella* Enteritidis). In 2010, 11.9 million typhoid fever cases were reported worldwide causing 129,000 deaths [3,4]. Typhoid fever incidence in sub-Saharan Africa is more than 100 cases per 100,000 person-years [3,4]. The global burden of non-typhoidal *Salmonella* is even higher, with 3.4 million cases reported worldwide in 2010, mainly affecting children < 5 years old and resulting in an estimated 680,000 deaths [5].

The pivotal role of human carriers in the transmission of typhoid fever is well-known [1,6,7]. In contrast, *Salmonella* Typhimurium and *Salmonella* Enteritidis infect a broad range of vertebrate animals and the reservoir of both serotypes is considered to be zoonotic [1]. However, there is evidence that both serotypes have genetically adapted to the human host [8], putting forward the possibility of a human reservoir. With regard to *Salmonella* intestinal carriage, it is defined as excretion of *Salmonella* in stool in absence of symptoms of infection; and those individuals who excrete the bacteria in their stool are called “*Salmonella* carriers” [9]. This situation has been described in typhoidal as well as non-typhoidal serovars. In contrast to typhoid fever, little is known about the frequency and duration of non-typhoidal *Salmonella* carriage after infection in sub-Saharan Africa [2].

Schistosomiasis is a neglected tropical disease caused by parasitic worms with freshwater snails as intermediate hosts. *Schistosoma mansoni* is one of the main species infecting humans; its adult worms live in the mesenteric capillaries [10]. Without treatment, *Schistosoma mansoni* infection may result in hepatosplenic inflammation and liver fibrosis [11].

There is clinical and experimental evidence towards a symbiotic relation between *Schistosoma* and *Salmonella* and associations have been described for different *Schistosoma* species and both Typhi and NTS serotypes. Clinical observations in patients from Egypt and Gabon showed that, in the presence of *Schistosoma* infection, relapsing or persistent *Salmonella* bacteremia or bacteriuria were only cured when concomitant anti-*Schistosoma* treatment was given [12–14]. Furthermore, hospital case series and cross-sectional studies from Nigeria and Egypt showed an overrepresentation of *Schistosoma* infections among patients with invasive salmonellosis [10,13] as well as an overrepresentation of *Salmonella* infection among *Schistosoma*-infected patients [15]. However, these studies had limitations. For example, patient groups were ill-defined and control groups were absent [10,12,13,15,16]. In addition, different reference tests were used including the Widal test which is based on typhoidal *Salmonella* (Typhi and Paratyphi) antibody detection and is not recommended as a diagnostic test in endemic settings [10,15]. Lastly, patients were given antibiotic treatments such as amoxicillin or chloramphenicol which are ineffective to prevent relapses [12,14,16]. These limitations preclude inter-study comparison and sound conclusions. Of note, a study in Malawi conducted in a cohort of HIV-infected patients, failed to demonstrate a clinical association between *Salmonella* and *Schistosoma* infections in terms of *Salmonella* recurrence and mortality [17].

Experimental studies demonstrated that *Salmonella* bacteria (mainly Paratyphi A and Typhimurium) were able to colonize adult *Schistosoma* worm by connecting their fimbrial protein (FimH) to a receptor located on the worm teguments [18–22]. Furthermore, increased morbidity and mortality were observed in co-infected mice and hamsters [18,20,23–27]. In addition, the physical association of *Salmonella* with *Schistosoma* worms was shown to increase the inhibitory concentrations of several classes of antibiotics [22]. Finally, alteration of the macrophage function in *S*. *mansoni*-infected mice was observed and this was related to growth and survival of *Salmonella* Typhimurium [28]. However, many of these experimental studies used very high doses of *Salmonella* injected intravenously, intraperitoneally or intracardially [18,21,24].

Despite the fact that well-designed epidemiological studies on *Salmonella-Schistosoma* coinfections are lacking, there is concern that concurrent *Schistosoma* infection may interfere with effectiveness of typhoid vaccines [29] and by extension with the future non-Typhoidal *Salmonella* vaccines.

In the Democratic Republic of Congo (DRC), located in Central-Africa, schistosomiasis and invasive *Salmonella* infections are endemic [30–35]. The main objective of the present study was to assess the prevalence of *Salmonella* intestinal carriage in a *Schistosoma mansoni* endemic community, and to assess a possible association between both pathogens. Additional objectives were to assess the serotype distribution of the intestinal *Salmonella* isolates and their genetic relatedness with invasive (blood culture) isolates.

## Methods

### Ethics statement

The study protocol was approved by the Institutional Review Board at the Institute of Tropical Medicine in Antwerp (Reference 852/12), the ethical committee of University of Antwerp (Reference 12/50/423) and the ethical committee of the Public Health School of Kinshasa, in the Democratic Republic of the Congo (References ESP/CE/022B/14 and ESP/CE/022C/2015). The study team obtained authorizations from the DRC ministry of health and the local authorities of the Kongo Central province. Before starting, the aim of the study and its benefits were explained to the community. All eligible adult participants were asked for written informed consent; children between 15 and 18 years old of age were additionally asked for written assent. For children under 15 years old, written informed consent from the legal representative was obtained.

### Study site

**Fig 1:**
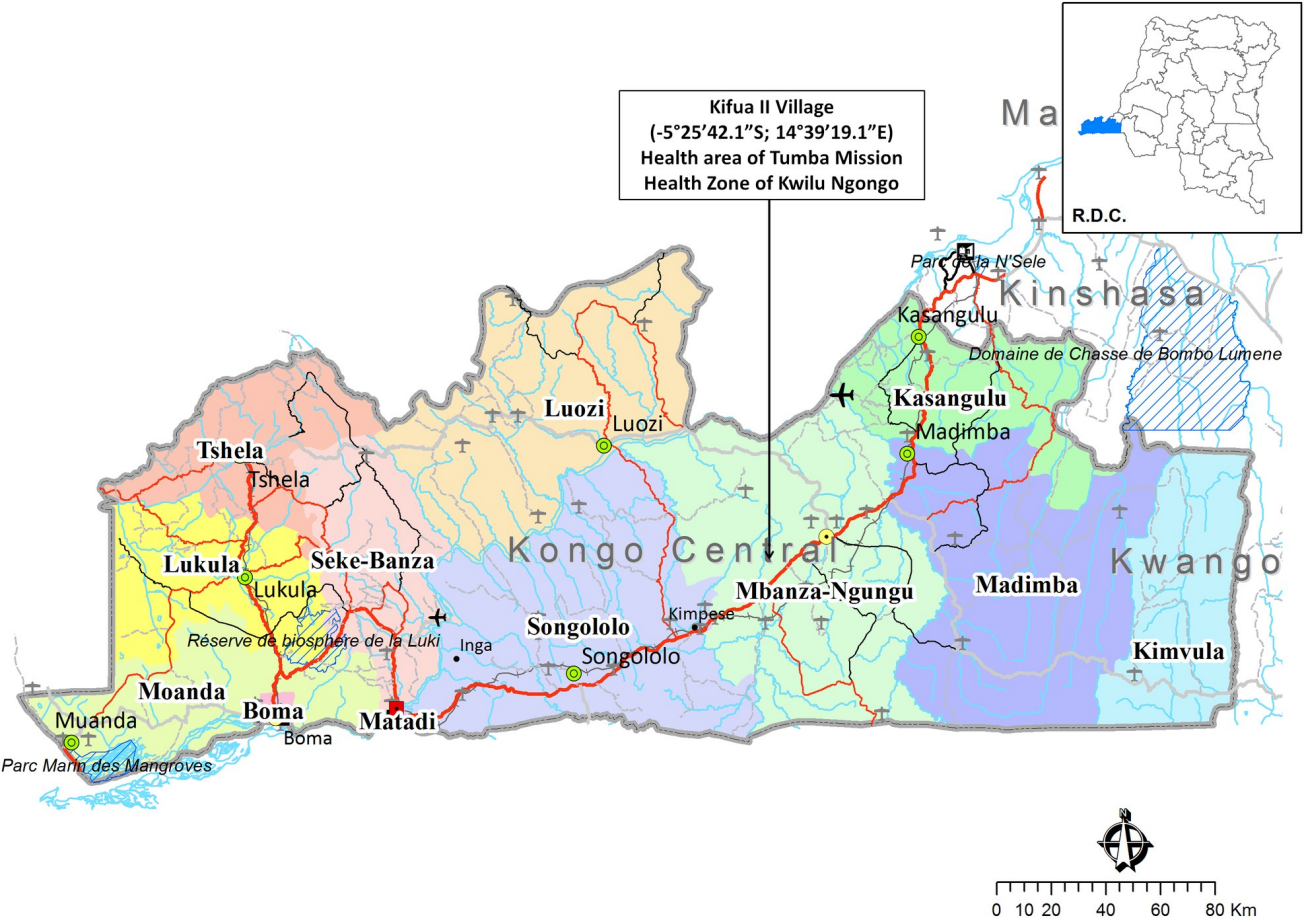
The province of Kongo Central in the Democratic Republic of Congo. Kifua II village, health area of Tumba Mission, Health Zone of Kwilu Ngongo. https://www.caid.cd/graphics/province/12_Kongo-Central.png.

The province of Kongo Central in DRC reported invasive *Salmonella* infections decades ago [36] but also more recently [33,37]. The area is also endemic for schistosomiasis with a predominance of *Schistosoma mansoni* infection [35]. After the assessment of six Health Reference Zones in the Province of Kongo Central during site visits, the village of Kifua II (-5°25´42.1”S; 14°39´19.1”E) situated in the health area of Tumba Misson and in the Health Zone of Kwilu Ngongo was selected as the study site (Fig 1). The study area extended over a surface of 196,560.90 m^2^ and had a population of about 1,400 inhabitants. The specific prevalence of HIV in the study population was not known however, in DRC the endemicity of HIV is less than 1% [38]. Prior to our study, no mass drug administration for the control of schistosomiasis had taken place. Kifua II is easily accessible by car, allowing for timely transport of stool samples to the laboratory of the reference hospital of Kimpese (Institut Médico-Evangélique of Kimpese, IME-Kimpese) at 35 km distance.

### Study design

This study was conducted from November 2015 to March 2016, during the rainy season, which is expected to have the highest number of *Salmonella* infections. It was embedded in an eco-epidemiological cross-sectional study on helminth co-infections and related morbidity patterns in a rural area of DRC. All inhabitants living for more than 1 year in Kifua II village and being more than 1 year old were eligible and were invited to participate in the study. The study team went to the village early morning to reach participants before they departed for field work. After giving informed consent, each participant was asked to give two consecutive stool samples within 1 week. Participants (or their caretakers) were also interviewed about demographics and recent history of fever (≤ 14 days), diarrhea (24 hours before enrollment) and antibiotic treatment (≤ 48 hours before enrollment).

### Detection of *Schistosoma mansoni* infection

Within 2 hours upon arrival at the laboratory of IME-Kimpese, stool samples were processed by the Kato Katz method (2 slides of 25 mg of fecal material for each stool sample, with a maximum of 4 slides per participant) and within 24 hours, slides were microscopically examined for the presence and number of *Schistosoma mansoni* eggs. The sum of the egg count from 4 slides was multiplied by 10 to reach one gram of stool. The *Schistosoma mansoni* infection intensity was expressed as the number of eggs detected per gram of feces (EPG). According to WHO guidelines, *Schistosoma* infection intensity was categorized as light, moderate or heavy at egg counts of 1–99 EPG, 100–399 EPG or ≥ 400 EPG, respectively [39].

One dose of 40mg/kg of praziquantel was administered to all inhabitants of the community at the end of the study.

### Detection of *Salmonella* carriage

Within 2 hours after sample reception at Kifua II health center, approximately 1 gram of each stool sample was suspended into 10 ml of selenite broth (BD Difco, Becton Dickinson and Company, Franklin Lakes, New Jersey). After that, all samples were transported to the laboratory of IME-Kimpese. Upon arrival, the selenite broth tubes were incubated for 18 hours at 35°C, then, 10 μl was sub-cultured on each of two plates of *Salmonella*-*Shigella* agar (SS) (Lab M Limited, Lancashire, United Kingdom). After incubation (18–24 hours at 35°C), colonies suspected for *Salmonella* (lactose negative with H_2_S positive center) were sub-cultured on Kligler Iron Agar (KIA) (Lab M Limited) for 18–24 hours at 35°C. Per suspected colony type, two colonies were sub-cultured on KIA with a maximum of four colony types per SS agar plate. In case of no growth on the SS agar, the plates were read after another 24 hours of incubation. Bacterial isolates from KIA tubes showing a pattern suggestive of *Salmonella* (glucose fermentation, lactose negative, H_2_S positive) were inoculated in a gallery consisting of three disk-based biochemical tests (DiaTabs, Rosco, Taastrup, Denmark): ortho-nitrophenyl galactopyranoside (ONPG), urease and lysine decarboxylase (LDC) combined with indole production. Isolates with a reaction pattern compatible with *Salmonella* (ONPG negative, urease negative, LDC positive and indole negative) were stored on 2-ml tubes with Trypticase Soya Agar (TSA, OXOID, Basingstoke, United Kingdom) and shipped to the National Institute of Biomedical Research (INRB, Kinshasa, DRC) and the Institute of Tropical Medicine (ITM, Antwerp, Belgium) for confirmation, serotyping (Vision^TM^, Pro-lab Diagnostics Inc., Richmond Hill, Ontario, Canada) and antibiotic susceptibility testing (AST). AST was performed by disk diffusion (Neo-Sensitabs, Rosco) according to the Clinical and Laboratory Standards Institute (CLSI) M100-S27 criteria (40). For ciprofloxacin and azithromycin, minimal inhibitory concentration (MIC) values were determined using the E-test macromethod (bioMérieux, Marcy L’Etoile, France) with breakpoints according to the CLSI guideline [40]. Multidrug resistance (MDR) was defined as co-resistance to the three first-line antibiotics (ampicillin, chloramphenicol and trimethoprim-sulfamethoxazole) [41].

It was decided not to treat *Salmonella* Typhi intestinal carriers with antibiotics given the (i) high endemicity of typhoid fever in sub-Saharan Africa (with a consequently less important role of household intestinal carriers in transmission) [46] and the (ii) high doses/long period of required ciprofloxacin treatment combined with its safety profile [47]. Likewise, antibiotic treatment was not installed for NTS carriers, with the additional argument that stool shedding of NTS is mostly of short duration [48,49].

### *Salmonella* multi-locus variable-numbers tandem repeat analysis (MLVA)

*Salmonella* Typhimurium (n = 4) and *Salmonella* Enteritidis isolates (n = 5) were sent to the Institute of Public health (IPH, Brussels, Belgium) for multi-locus variable-numbers tandem repeat analysis (MLVA) as previously described [42–44]. MLVA profiles were attributed based on the number of tandem repeats on five loci (SENTR7, SENTR5, SENTR6, SENTR4, SE3 for *Salmonella* Enteritidis and STTR9, STTR5, STTR6, STTR10, STTR3 for *Salmonella* Typhimurium isolates). In addition, *Salmonella* Typhimurium and *Salmonella* Enteritidis isolates (n = 19 and n = 10 respectively) recovered from a blood culture surveillance performed at the referral hospital of Kisantu (at 66 km from Kifua II) were used for comparison of MLVA types; isolates were representative for time (period September 2015 –December 2016) and antibiotic susceptibility profile. MLVA patterns were compared for genetic relatedness: a Typhimurium cluster was defined as isolates with variation in none or one of the rapidly changing loci (STTR5, STTR6 and STTR10) and no variation in the stable loci (STTR3 or STTR9) [45]; an Enteritidis cluster was defined as isolates with variation in none or one of the five loci [43]. A major MLVA type was defined as MLVA profiles comprising more than one *Salmonella* isolate.

### Data collection and analysis

Field data and laboratory results were registered into case report forms and laboratory registers.

Data entry and analysis were done using Excel 2016 (Microsoft, Richmond, US). Duplicate *Salmonella* isolates from consecutive samples were removed from analysis. Data were characterized by frequencies, proportions, medians, interquartile range (IQR) and 95% confidence interval (95% CI). Chi-square test or Fisher exact test were used to test the association between *Salmonella* and *Schistosoma mansoni* infection. Results were considered significant when the p-value was < 0.05.

## Results

### Study population

In the village Kifua II, there were 1,304 eligible inhabitants among whom 196 (15%) did not participate to the study. Main reasons of non-participation were absence from the site (113/196) and refusal (55/196). Among 1,136 inhabitants who gave consent, 1,108 provided at least one stool sample. Their median (IQR) age was 15 (7–36) years, with a male-to-female rate of 1:1.1. Of them, 865 (78.0%) provided a second stool sample (S1 Fig). Of the participants answering the different questions of the survey, 3.2% (36/1,108) reported use of antibiotics within 48 hours before enrollment, 2.6% (29/1,108) and 21.3% (236/1,108) reported a history of diarrhea and fever within 14 days before enrollment, respectively. *Salmonella* carriage was neither associated with recent history of fever (8/38 versus 222/1,031, p = 0.943) nor with diarrhea (1/38 versus 28/1,030 p = 0.974) and none of the *Salmonella* carriers had taken antibiotics within 48 hours before enrollment.

### *Schistosoma mansoni* infection

More than half of the participants (51.2%, 567/1,108; 95% CI: 48.2–54.1) were *Schistosoma mansoni* positive; infection intensities were light, moderate and heavy in respectively 51.3%, 30.5% and 18.2% (Table 1).

**Table 1:** Number of participants with *Salmonella* intestinal carriage and *Schistosoma mansoni* infection in Kifua II, including the distribution of *Schistosoma mansoni* egg loads for *Schistosoma mansoni* positive participants.

	*Salmonella* carrier	No *Salmonella* carrier	Total	*P*-value
***Schistosoma mansoni* negative**	14 (2.6%)*	527 (97.4%)*	541 (48.8%)**	0.132
***Schistosoma mansoni* positive**	24 (4.2%)*	543 (95.8%)*	567 (51.2%)**	
Light egg load	11 (3.8%)*	280 (96.2%)*	291 (51.3%)^†^	0.553^¶^
Moderate egg load	4 (2.3%)*	169 (97.7%)*	173 (30.5%)^†^	0.012^¶¶^
Heavy egg load	9 (8.7%)*	94 (91.3%)*	103 (18.2%)^†^	0.002^¶¶¶^
**Total**	38 (3.4%)*	1070 (96.6%)*	1108	

*Percentages for the total of each row

** Percentages for the total number of participants

^†^Percentages for the total number of *Schistosoma mansoni* positive participants

^¶^p-value of proportions of *Salmonella* carriers among persons with light *Schistosoma* infection and those with moderate infection.

^¶¶^p-value of proportions of *Salmonella* carriers among persons with heavy *Schistosoma* infection and those with light and moderate infection combined.

^¶¶¶^p-value of proportions of *Salmonella* carriers among persons with heavy *Schistosoma* infection and those not infected by *Schistosoma*.

### *Salmonella* intestinal carriers

A total of 38 participants were detected as *Salmonella* carriers, representing an overall carriage rate of 3.4% (95% CI: 2.5–4.7) of the study population. The number of *Salmonella* isolates among the first and second samples was 25/1,108 (2.3%) and 14/865 (1.6%) respectively. One patient (male, 56 years old) had a stool sample grown with *Salmonella* Kisarawe in both samples (second sample submitted 1 day after the first sample). *Salmonella* carriage was neither associated with recent history of fever (8/38 versus 222/1,031; p = 0.943) nor with diarrhea (1/38 versus 28/1,030; p = 0.974) and none of the *Salmonella* carriers had taken antibiotics within 48 hours before enrollment.

### *Salmonella* intestinal carriage in relation to *Schistosoma mansoni* infection

Among the 38 *Salmonella* carriers, 24 participants were coinfected with *Schistosoma mansoni*, representing 2.2% (95% CI: 1.5–3.2) of the total study population. The proportion of *Salmonella* carriage among *Schistosoma* infected participants tended to be higher (4.2%, 24/567; 95% CI: 2.9–6.2) compared to *Schistosoma* non-infected participants (2.6%, 14/541; 95% CI: 1.6–4.3) but this difference did not reach statistical significance (p = 0.132). Moreover, the proportion of *Salmonella* carriers among *Schistosoma*-infected participants with heavy infection intensity (8.7%, 9/103; 95% CI: 4.7–15.8) was significantly higher than the proportion among those with light and moderate infection intensities combined (3.2%, 15/464; 95% CI: 1.9–5.3; p = 0.012). It was also higher than the proportion of *Salmonella* carriers among the *Schistosoma* negative participants (2.6%, 14/541; p = 0.002) (Table 1).

### Serotype distribution of *Salmonella* isolates

Among the 38 *Salmonella* isolates (Table 2), 15 different serotypes were found of which *Salmonella* Stanleyville and *Salmonella* Enteritidis represented five isolates each. *Salmonella* Typhimurium and Rubislaw represented four isolates each. *Salmonella* Typhi was not found. There were two households in which two participants were carrier of the same serotype (*Salmonella* Typhimurium and *Salmonella* Kisarawe respectively), otherwise no particular clusters were seen. MDR was only observed among *Salmonella* Typhimurium and S*almonella* Enteritidis (4/4 isolates and 1/5 isolates respectively). All isolates were susceptible to ciprofloxacin, tetracycline, ceftriaxone and azithromycin.

**Table 2:** Distribution of *Salmonella* isolates according to serotype.

Serotypes	Number
*Salmonella* Enteritidis	5
*Salmonella* Stanleyville	5
*Salmonella* Typhimurium	4
*Salmonella* Rubislaw	4
*Salmonella* Brive	3
*Salmonella* Virchow	3
*Salmonella* sp.	3
*Salmonella* Kisarawe	2
*Salmonella* Urbana	2
*Salmonella* Aberdeen	1
*Salmonella* Akanji	1
*Salmonella* Essen	1
*Salmonella* Kasenyi	1
*Salmonella* Livingstone	1
*Salmonella* Sanga	1
*Salmonella* Teshie	1
**Total**	**38**

There was no association between *Salmonella* serotype and *Schistosoma* co-infection. Although the numbers were too small to trace associations, it was observed that three out of five MDR isolates occurred among children < 5 years old. Likewise, *Salmonella* Typhimurium tended to occur more among children < 15 years old (3/4 isolates), whereas non-MDR *Salmonella* Enteritidis occurred more frequently among adults (3/4 isolates).

### MLVA typing of intestinal and blood culture *Salmonella* Typhimurium and Enteritidis isolates

**Fig 2:**
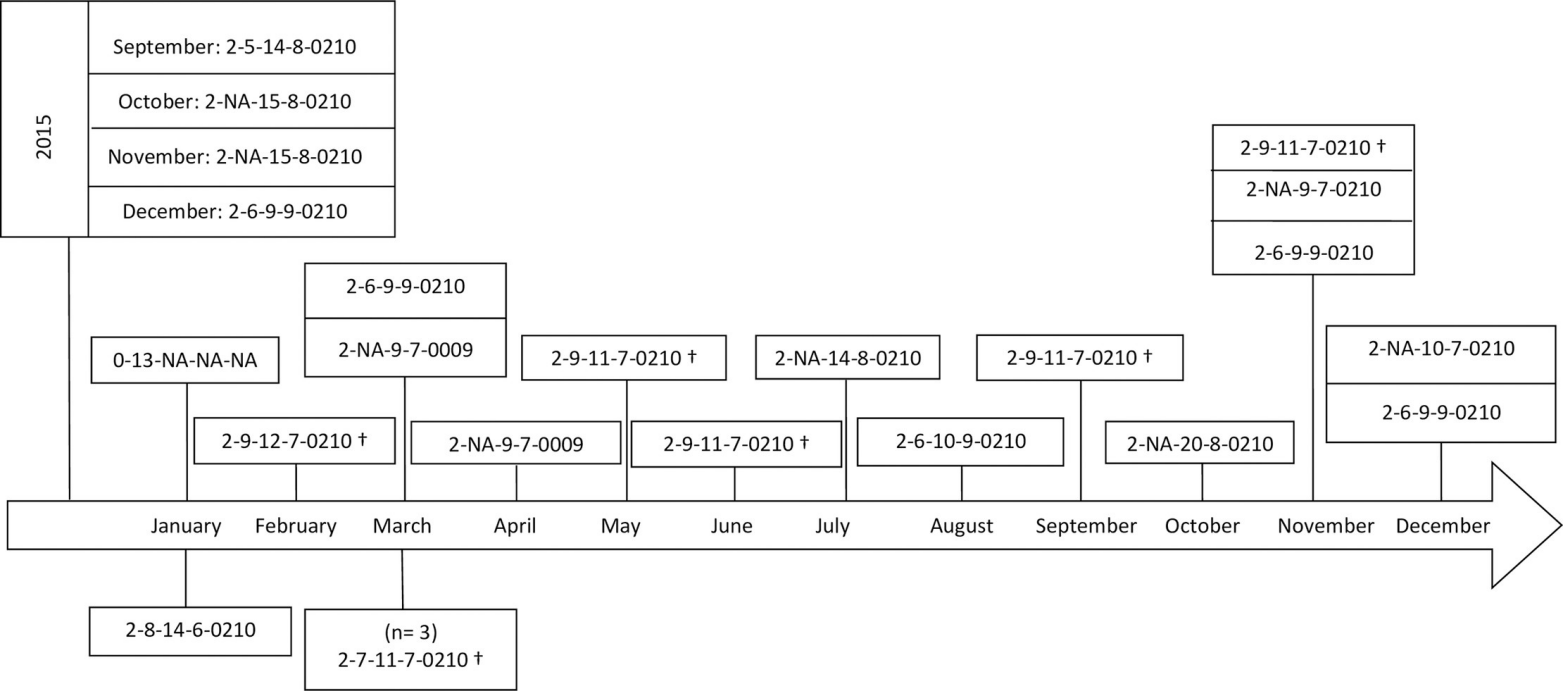
Multi-locus variable-number tandem repeat analysis (MLVA) of 19 *Salmonella* Typhimurium invasive isolates (blood cultures, above the timeline) versus four *Salmonella* Typhimurium intestinal isolates (stool culture, below the timeline). Position of the isolates along the timeline represents the time of isolation in 2015 or 2016. Series of 5 numbers represent 5 loci on which each number corresponds to the numbers of alleles amplified and “NA” corresponds to a locus on which no allele has been amplified. Symbol “†” denotes highly related MLVA types among intestinal and invasive isolates.

**Fig 3:**
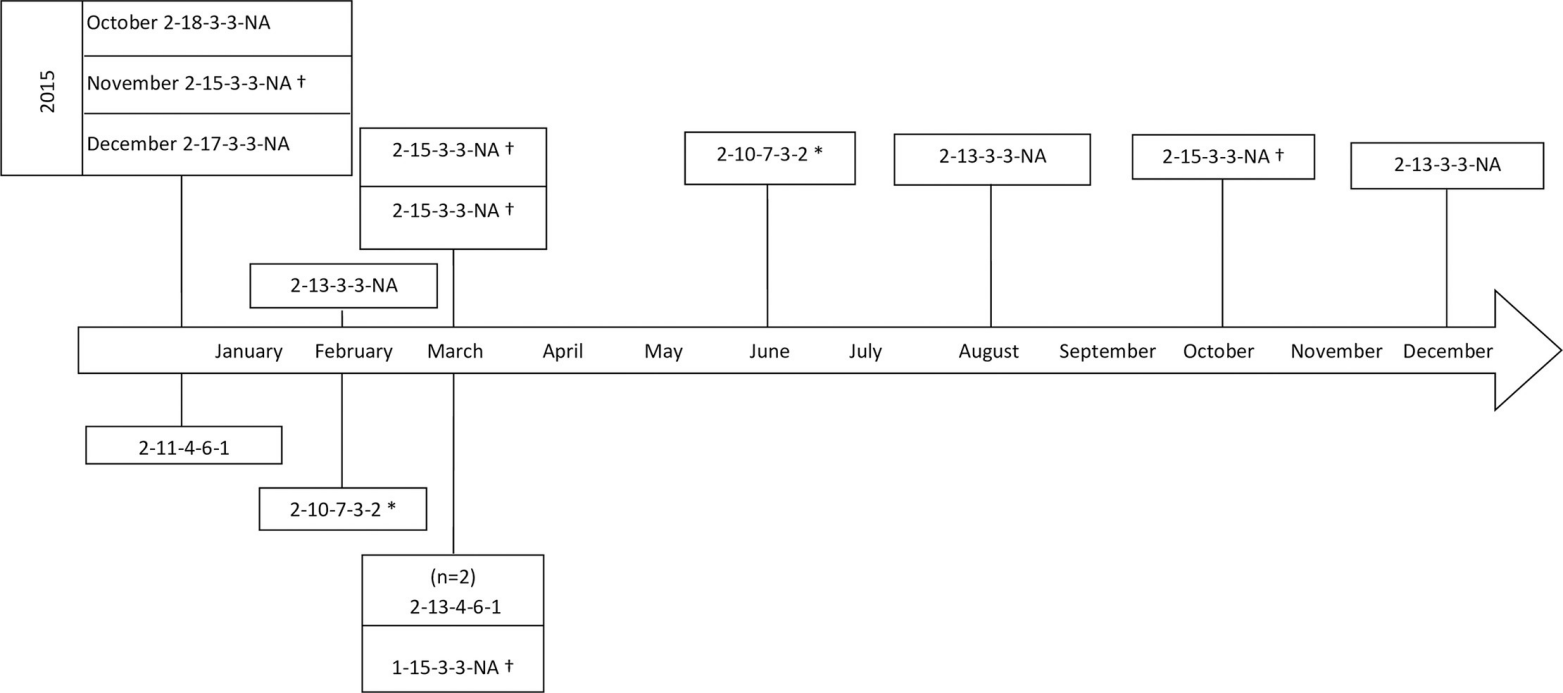
Multi-locus variable-number tandem repeat analysis (MLVA) of 10 *Salmonella* Enteritidis isolated from blood (above the timeline) versus five *Salmonella* Enteritidis isolated from stool (below the timeline). Position of the isolates represents the time of isolation in 2015 or 2016. Series of 5 numbers represent 5 loci on which each number corresponds to the numbers of alleles amplified and “NA” corresponds to a locus on which no allele has been amplified. Symbol “†” denotes highly related MLVA types among intestinal and invasive isolates. Symbol “*” denotes identical MLVA types among intestinal and blood culture isolates.

The four *Salmonella* Typhimurium intestinal isolates were compared to 19 blood culture isolates. Six major MLVA types and four other MLVA types (comprising only one isolate) were observed. Three out of four intestinal Typhimurium isolates shared similar MLVA types with blood culture isolates (Fig 2). The five *Salmonella* Enteritidis intestinal isolates were compared to 10 blood culture isolates and yielded three major MLVA types. Two out of five intestinal Enteritidis isolates shared identical or similar MLVA types with blood culture isolates (Fig 3).

## Discussion

The present study was conducted in a *Schistosoma mansoni* endemic community in a rural area in DRC. Results showed a prevalence of intestinal *Salmonella-Schistosoma* co-infections of 2.2% in the study population. The percentage of *Salmonella* intestinal carriage tended to be higher among *Schistosoma*-infected than among *Schistosoma* non-infected participants (4.2% versus 2.6%). Furthermore, the proportion of *Salmonella* carriage was significantly higher among participants with a heavy *Schistosoma mansoni* infection intensity (8.7%) compared to those with light and moderate infection intensities (3.2%). Among 38 *Salmonella* isolates (overall carriage rate of 3.4%), Enteritidis and Typhimurium serotypes were found (n = 5 and n = 4 respectively), most of which had MLVA types identical or similar to those from blood cultures.

The present findings add to the evidence of an association between *Schistosoma mansoni* infection and *Salmonella* intestinal carriage. The fact that the association between *Schistosoma* and *Salmonella* mainly occurred among the heavy infection intensity group fits with clinical observations of co-infections in long-standing or serious *Schistosoma* infections [14,50].

Previous community-based studies among asymptomatic participants reported a *Schistosoma-Salmonella* association in two cross-sectional surveys in Nigeria, reporting 4.4% and 5.4% of *Schistosoma mansoni–Salmonella* co-infected participants [13,51]. Two studies from Sudan reported 30.9% and 60.0% of *Salmonella* Typhi/Paratyphi excretion in urine or stool among *Schistosoma*-infected patients [52,53]. However, in both studies, control groups were not assessed for *Salmonella* carriage and in none of the aforementioned studies were selection and representativeness of the participants described.

In our study, the overall *Salmonella* carriage rate was 3.4%, and only non-typhoidal *Salmonella* were isolated. Few studies from sub-Saharan Africa have addressed *Salmonella* intestinal carriage, and most have addressed food handlers and related professions [54]. In recent community surveys in Guinea-Bissau and Senegal, *Salmonella* intestinal carriage rate was 2.4% and 1.0% respectively [54]. These lower proportions may be explained by the fact that in these surveys only a single stool sample per participant was assessed as well as by a difference in access to safe water supply and sanitation.

Most intestinal *Salmonella* isolates belonged to serotypes different from the invasive Typhimurium and Enteritidis serotypes [33]. However, both invasive serotypes (n = 9) accounted for a quarter of the intestinal isolates and represented 0.8% (9/1,108) of the overall study population. Most of them had MLVA types identical or similar to those of the blood culture isolates obtained in the same period from a hospital at 66 km distance. It may be expected that, if the blood culture surveillance had been conducted closer to Kifua II, even a better concordance of intestinal and invasive Typhimurium and Enteritidis MLVA types would have been observed. This observation adds to the evidence of the human nature (reservoir and potentially transmission) of the invasive Typhimurium and Enteritidis NTS serotypes.

The present study has some limitations. First, for logistical reasons only a maximum of two successive stool samples per patient were requested. In view of the intermittent excretion of *Salmonella* organisms, three successive stool samples would have been optimal [49,55] and could have yielded higher proportions of *Salmonella* carriage and *Schistosoma mansoni* infections. However, obtaining three successive samples in field studies may be challenging due to participant reluctance [55]. Of note, previous studies assessing *Schistosoma-Salmonella* interactions were mostly based on only one stool sample per participant [10,13,51]. Likewise, *Schistosoma* eggs excretion may have variations and the proportion of light *Schistosoma* infections could have been higher, as the Kato Katz method tends to be false-negative at low egg loads [55]. Immunological methods such as circulating cathodic antigen (CCA) detection in urine are promoted for the diagnosis of *Schistosoma mansoni* infection in endemic communities. The CCA-point-of-care test is related to the intensity of infection but does not express egg load [56,57]. In addition, it would not have provided better results than microscopy, as it may be false negative in light infections and requires more than one sample for optimal sensitivity [55].

A second limitation of this study is the small number amount of *Salmonella* carriers, which did not allow to reach statistical significance in the assessment of the overall association between *Salmonella* carriage and *Schistosoma* infection. The third limitation is that we did not assess possible associations between *Salmonella* and other species of *Schistosoma* such as *Schistosoma haematobium* [50].

A strength of the present study is that it is, to our knowledge, the first population-based study on the association of *Salmonella* intestinal carriage with *Schistosoma mansoni* infections in Central Africa. Moreover, sample work-up including sample transport conditions and delays were well controlled and complied with the prescribed norms [58]. Furthermore, unlike previous studies, the association between *Schistosoma mansoni* infection intensity and *Salmonella* carriage was investigated as well as the distribution of *Salmonella* serotypes and their genetic relatedness with invasive isolates.

The impact of this study for the control of invasive salmonellosis may be summarized as follows: even among heavy *Schistosoma mansoni* infections, the proportion of invasive *Salmonella* serotypes (Typhimurium and Enteritidis) was still low. Based on the present findings, *Schistosoma mansoni* infection is not expected to constitute a barrier to immunization of populations by *Salmonella* vaccination as mentioned in a recent review [29]. Further studies are needed to confirm the present findings for other *Schistosoma* species and *Salmonella*-infected patients (bacteremia and bacteriuria).

## Supporting information

S1 Strobe Checklist (DOC)

S1 Fig (PDF)
