# Supplementary material for: Non-typhoidal Salmonella intestinal carriage in a Schistosoma mansoni endemic community in a rural area of the Democratic Republic of Congo
Source: PLoS Negl Trop Dis. 2020 Feb 21;14(2):e0007875. doi: 10.1371/journal.pntd.0007875 (PMC7034803; doi:10.1371/journal.pntd.0007875)
Supplement: S1 Strobe Checklist — (DOC) [file pntd.0007875.s001.doc]

STROBE Statement—Checklist of items that should be included in reports of ***cross-sectional studies***

|  | Item No | Recommendation |
| --- | --- | --- |
| **Title and abstract** | 1 | (*a*) Indicate the study’s design with a commonly used term in the title or the abstract  *Done: in the “Abstract line 32” as cross-sectional community-wide study* |
|  |  | (*b*) Provide in the abstract an informative and balanced summary of what was done and what was found  ***We describe what was done in the “Abstract line 33 – line 38”****: Stool samples were collected and analyzed for Salmonella intestinal carriage (culture) and Schistosoma mansoni infection (Kato Katz microscopy with determination of egg load). Salmonella Typhimurium and Enteritidis isolates were assessed for genetic similarity with blood culture isolates obtained during the same period in a neighboring hospital using multi-locus variable-numbers tandem repeat analysis (MLVA).*  ***We describe what was found in the “Abstract line 39 – line 49”****: A total of 1,108 participants were included (median age 15 years (IQR: 7-36), male-to-female ratio of 1:1.1). The overall prevalence of Schistosoma mansoni infection and non-typhoidal Salmonella carriage was 51.2% (95% CI: 48.2 – 54.1) and 3.4% (95% CI: 2.5 – 4.7) respectively, with 2.2% (95% CI: 1.5 – 3.2) of participants coinfected. The proportion of Salmonella carriage tended to be higher among Schistosoma mansoni infected participants compared to non-infected participants but this difference did not reach statistical significance (4.2% versus 2.6%, p = 0.132).* *However, the proportion of Salmonella carriage among participants with a heavy Schistosoma mansoni infection intensity was significantly higher compared to those with a light and moderate infection intensity (8.7% versus 3.2%, p = 0.012) and compared to Schistosoma mansoni negatives (8.7% versus 2.6%, p = 0.002). The 38 Salmonella isolates comprised five and four Enteritidis and Typhimurium serotypes respectively, the majority of them had MLVA types identical or similar to those observed among blood culture isolates.* |
| Introduction | | |
| Background/rationale | 2 | Explain the scientific background and rationale for the investigation being reported  ***Done: the paragraph 2*** *explains what is known about Salmonella reservoir and the interest of assessing non-typhoidal Salmonella carriage,* ***the paragraphs 4 and 5*** *report clinical and experimental* *evidences of the relation between Schistosoma and Salmonella* |
| Objectives | 3 | State specific objectives, including any prespecified hypotheses  ***The objectives are specified in the paragraph 6:*** *The main objective of the present study was to assess the prevalence of Salmonella intestinal carriage in a Schistosoma mansoni endemic community, and to assess a possible association between both pathogens. Additional objectives were to assess the serotype distribution of the intestinal Salmonella isolates and their genetic relatedness with invasive (blood culture) isolates.* |
| Methods | | |
| Study design | 4 | Present key elements of study design early in the paper  ***Done: In the paragraph 3 of the methods (study design section)*** “*The study was conducted from November 2015 to March 2016 and was embedded in an eco-epidemiological cross-sectional study on helminth co-infections and related morbidity patterns in a rural area of DRC. All inhabitants living for more than 1 year in Kifua II village and being more than 1 year old were eligible and were invited to participate in the study. After giving informed consent, each participant was asked to give two consecutive stool samples within 1 week. Participants (or their caretakers) were also interviewed about demographics and recent history of fever (≤ 14 days), diarrhea (24 hours before enrollment) and antibiotic treatment (≤ 48 hours before enrollment).”* |
| Setting | 5 | Describe the setting, locations, and relevant dates, including periods of recruitment, exposure, follow-up, and data collection  ***Done in paragraph 2 and 3****: study site and study design under Methods section* |
| Participants | 6 | (*a*) Give the eligibility criteria, and the sources and methods of selection of participants  ***Done in paragraph 3****: study design under Methods section* |
| Variables | 7 | Clearly define all outcomes, exposures, predictors, potential confounders, and effect modifiers. Give diagnostic criteria, if applicable  ***Done in paragraph 4, 5 and 6: (i)*** *Detection and definition of Schistosoma mansoni infection and intensity (egg load), (ii) Detection and definition of Salmonella carriage and serotype, (iii) Salmonella multi-locus variable numbers tandem repeat analysis under Methods section.* |
| Data sources/ measurement | 8* | For each variable of interest, give sources of data and details of methods of assessment (measurement). Describe comparability of assessment methods if there is more than one group  ***Done in paragraph 7:*** *Sample and data collection, shipment and analysis of samples and quality control under Methods section. Description of statistical methods.* |
| Bias | 9 | Describe any efforts to address potential sources of bias  ***Done in Methods, paragraph 2 and 3 (line 144 - 168)***  *- Bias through non-participating: 85% (1,108/1,304) of eligible persons participated to the study. breakdown see the breakdown of study population under supplementary information*  *- Bias in the prevalence of Schistosoma mansoni infection: the study site was chosen taking into account that no mass drug administration for the control of schistosomiasis had taken place prior to the study*  *- Salmonella isolation by stool culture: we have chosen as study site the village Kifua II which was easily accessible by car, allowing for timely transport of stool samples to the laboratory of the reference hospital of Kimpese. The village is representative for the health area.*  *- Bias by low sensitivity of stool culture compensated by (i) 2 successive samples and (ii) short transport times*  ***Done in Methods, paragraph 4 and 5:*** *samples were handled with respect of validated standardized operating procedures*  *Additionally, study team used to go to the village early in the morning to reach participants before they could go on field work. This was done to avoid any impact of participation rate on the prevalence of both Schistosoma mansoni infection and Salmonella carriage* |
| Study size | 10 | Explain how the study size was arrived at  ***Not applicable*** *(see Methods, paragraph 2): convenience sampling based on an existing helminth study without any preliminary data on beforehand - we included all eligible and consenting participants living in Kifua II village* |
| Quantitative variables | 11 | Explain how quantitative variables were handled in the analyses. If applicable, describe which groupings were chosen and why  ***Done in Methods, paragraph 8****: Data collection and analysis (grouping, confidence intervals, proportions)* |
| Statistical methods | 12 | (*a*) Describe all statistical methods, including those used to control for confounding  ***Done in Methods, paragraph 8****: Data collection and analysis* |
|  |  | (*b*) Describe any methods used to examine subgroups and interactions  ***Not applicable*** |
|  |  | (*c*) Explain how missing data were addressed  ***Not applicable*** |
|  |  | (*d*) If applicable, describe analytical methods taking account of sampling strategy |
|  |  | (*e*) Describe any sensitivity analyses  ***Not applicable*** |
| Results | | |
| Participants | 13* | (a) Report numbers of individuals at each stage of study—eg numbers potentially eligible, examined for eligibility, confirmed eligible, included in the study, completing follow-up, and analysed  ***Done in Results, paragraph 1****: Study population* |
|  |  | (b) Give reasons for non-participation at each stage  ***Done in the supporting information S1 Figure*** |
|  |  | (c) Consider use of a flow diagram  ***Done in the supporting information S1 Figure*** |
| Descriptive data | 14* | (a) Give characteristics of study participants (eg demographic, clinical, social) and information on exposures and potential confounders  ***Done in Results, paragraph 1****: Study population* |
|  |  | (b) Indicate number of participants with missing data for each variable of interest  ***Not applicable*** |
| Outcome data | 15* | Report numbers of outcome events or summary measures |
| Main results | 16 | (*a*) Give unadjusted estimates and, if applicable, confounder-adjusted estimates and their precision (eg, 95% confidence interval). Make clear which confounders were adjusted for and why they were included  ***Done in Results, paragraph 2, 3 and 4*** |
|  |  | (*b*) Report category boundaries when continuous variables were categorized  ***Not applicable*** |
|  |  | (*c*) If relevant, consider translating estimates of relative risk into absolute risk for a meaningful time period  ***Not applicable*** |
| Other analyses | 17 | Report other analyses done—eg analyses of subgroups and interactions, and sensitivity analyses  ***Not applicable*** |
| Discussion | | |
| Key results | 18 | Summarise key results with reference to study objectives  ***Done in Discussion, paragraph 1*** |
| Limitations | 19 | Discuss limitations of the study, taking into account sources of potential bias or imprecision. Discuss both direction and magnitude of any potential bias  ***Done in Discussion, paragraph 6*** |
| Interpretation | 20 | Give a cautious overall interpretation of results considering objectives, limitations, multiplicity of analyses, results from similar studies, and other relevant evidence  ***Done in Discussion, paragraph 2, 3 and 4*** |
| Generalisability | 21 | Discuss the generalisability (external validity) of the study results  ***Done in Discussion, paragraph 7 and 8*** |
| Other information | | |
| Funding | 22 | Give the source of funding and the role of the funders for the present study and, if applicable, for the original study on which the present article is based  ***Done in the financial disclosure*** |

*Give information separately for exposed and unexposed groups.

**Note:** An Explanation and Elaboration article discusses each checklist item and gives methodological background and published examples of transparent reporting. The STROBE checklist is best used in conjunction with this article (freely available on the Web sites of PLoS Medicine at http://www.plosmedicine.org/, Annals of Internal Medicine at http://www.annals.org/, and Epidemiology at http://www.epidem.com/). Information on the STROBE Initiative is available at www.strobe-statement.org.
