# Supplementary figures and images for: Non-typhoidal Salmonella intestinal carriage in a Schistosoma mansoni endemic community in a rural area of the Democratic Republic of Congo
Source: PLoS Negl Trop Dis. 2020 Feb 21;14(2):e0007875. doi: 10.1371/journal.pntd.0007875 (PMC7034803; doi:10.1371/journal.pntd.0007875)

## Study population

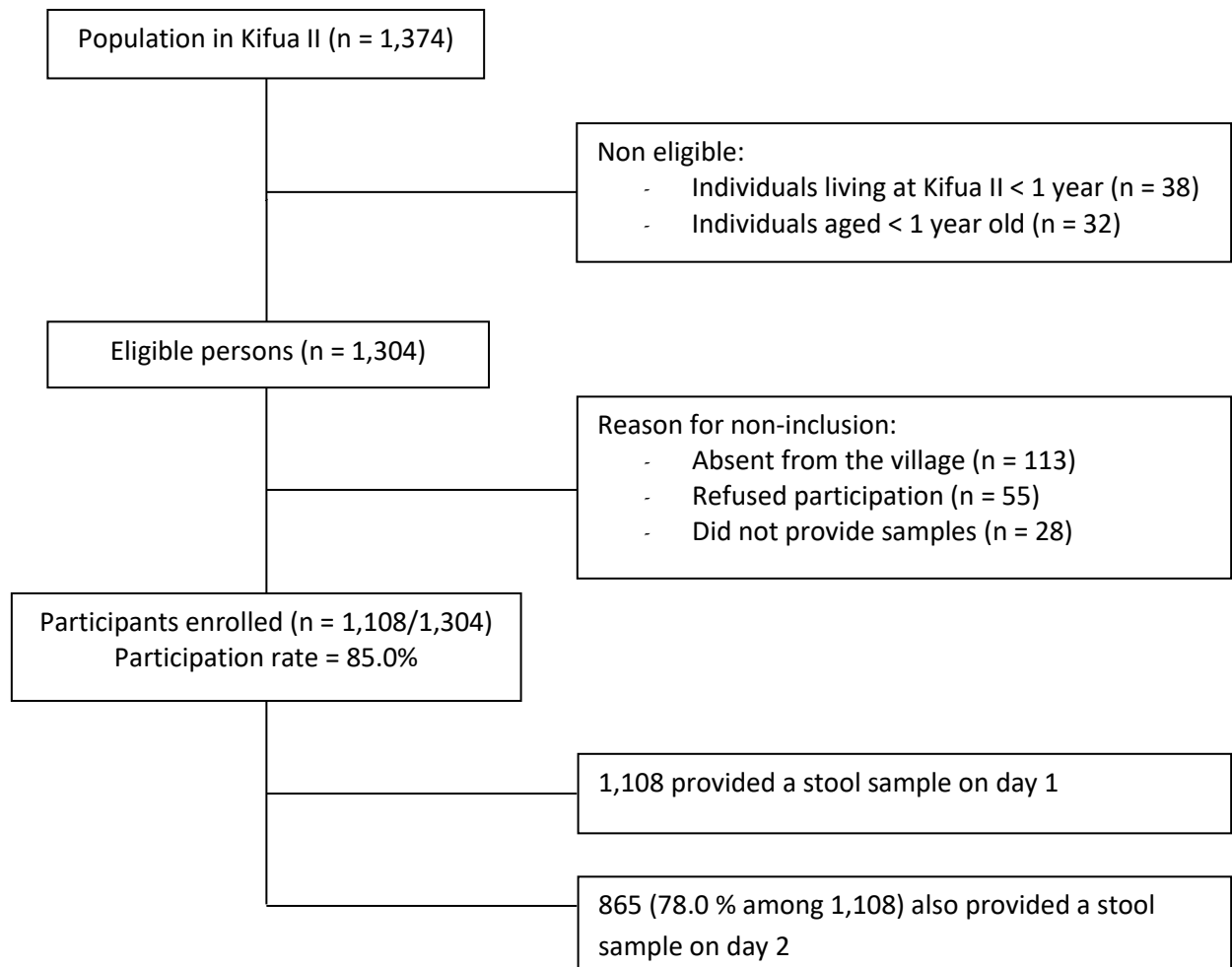

Supplement: S1 Fig — (PDF) [file pntd.0007875.s002.pdf]
